# Assessing Postnatal Immunisation Services in a Low-Resource Setting: A Cross-Sectional Survey

**DOI:** 10.3390/healthcare13121389

**Published:** 2025-06-11

**Authors:** Alhassan Sibdow Abukari, Rejoice Gaddah, Emmanuella Vincentia Ayivor, Ibrahim Sadik Haruna, Emmanuel Kwame Korsah

**Affiliations:** 1 Department of Maternal and Child Health, School of Nursing & Midwifery, University of Ghana, Legon, Accra P.O. Box LG 25, Ghana; asabukari@ug.edu.gh; 2 Department of Nursing, Wisconsin International University College, North Legon, Accra P.O. Box LG 751, Ghana; 11623391@wiuc-ghana.edu.gh (R.G.); 11621684@wiuc-ghana.edu.gh (E.V.A.); 3 University of Ghana Hospital, Legon, Accra P.O. Box LG 25, Ghana; isadik208@gmail.com; 4 Division of Clinical Associates, Department of Family Medicine, Faculty of Health Sciences, University of the Witwatersrand, Johannesburg WITS 2050, South Africa

**Keywords:** postnatal immunisation, immunisation adherence, vaccine accessibility, vaccine hesitancy, low-resource settings, neonatal health, immunisation coverage, confirmatory factor analysis, community-based initiatives, Socio-Ecological Model

## Abstract

**Background/Objectives:** Postnatal immunisation is critical for maternal and child health, particularly in low-income countries. Despite international efforts, maternal awareness and access to care remain limited. Understanding the drivers behind postnatal immunisation services is critical for improving neonatal and maternal healthcare interventions. **Methods:** A tertiary healthcare facility’s postpartum mothers who were seeking immunisation services participated in a prospective cross-sectional electronic survey. Convenience sampling was used to select respondents, who then answered a structured electronic questionnaire intended to obtain information on immunisation practices. To evaluate important trends and correlations, data was analysed using both descriptive and inferential statistics. A factor analysis was performed using the principal component analysis method, eigenvalue criteria, communalities, and confirmatory factor analysis. The study adhered to the STROBE guidelines. **Results:** We found that postnatal mothers’ good immunisation practices were influenced by their adherence to immunisation schedules (% variance: 56.407; Eigenvalue: 7.33), and significant satisfaction with communication (% variance: 8.338; Eigenvalue: 1.084); giving a cumulative variance explained of 64.745% of the total variance of variables under study. However, suboptimal practices include limited resource availability, poor record maintenance, insufficient support for side effects, a lack of support from healthcare providers, and a decline in immunisation recommendations, all of which had Eigenvalue <1 and insignificant percentage of variance contribution to the total variance explained. We developed a two-factor model of postnatal immunisation practices, focusing on adherence and effective communication. The model showed high loadings and adequate fit indices (χ^2^(34) = 91.333, *p* < 0.001; CFI = 0.945; TLI = 0.927; RMSEA = 0.071; SRMR = 0.042), good evidence of internal consistency (*α* = 0.823–0.877), and composite reliability (*ω* = 0.832–0.877). **Conclusions:** The study recommends a comprehensive approach to increasing newborn vaccine coverage, which includes health education, improved service delivery, and culturally sensitive communication strategies. Future research should focus on digital health interventions, community-based initiatives, and policy-driven postnatal care.

## 1. Introduction

Postnatal immunisation is a fundamental component of neonatal healthcare; it involves the administration of antigens to protect newborns from life-threatening diseases and contribute to global child survival efforts. Immunisation is a highly effective public health intervention that reduces infant mortality and prevents vaccine-related infections [1,2]. Postnatal immunisations, which primarily benefit newborns, are critical for providing early protection against infectious diseases. These services are an essential part of neonatal healthcare, with vaccines administered on a regular basis to protect newborns from preventable illnesses like tuberculosis, polio, diphtheria, pertussis, tetanus, and measles [3,4,5]. Despite their established importance, barriers such as a lack of awareness, insufficient healthcare infrastructure, vaccination hesitancy, and accessibility issues continue to impede optimal postnatal immunisation coverage in many regions, particularly in low-resource settings. To maximise the benefits of postnatal immunisation for both mothers and their infants, this quantitative survey was designed to assess the postnatal immunisation practices of mothers in a postnatal clinic at a tertiary facility in a low-resource setting.

Postnatal immunisation services provide newborns with necessary vaccines to protect them from infectious diseases in the early months of life. These vaccinations are inoculated on a predetermined schedule through national immunisation programmes such as the Expanded Programme on Immunisation (EPI), which ensures systematic administration during the first year of life [6]. Some vaccines, such as the Bacillus Calmette-Guérin (BCG) for tuberculosis and the oral polio vaccine (OPV), are given immediately after birth, while others, such as the diphtheria, pertussis, tetanus (DPT), and measles vaccines, are given at predetermined intervals [6]. These vaccines play an important role in increasing active, long-lasting immunity, preventing outbreaks, and lowering neonatal and infant mortality.

The success of immunisation programmes has been well documented. Experts estimate that timely vaccinations avert millions of child deaths annually [3,4]. Countries with well-established immunisation schedules practised in the EPI have seen significant improvements in child health, leading to higher survival rates [6]. The benefits go beyond individual protection, as high immunisation coverage promotes herd immunity, which indirectly protects unvaccinated individuals in the community (community immunity) [6]. This benefit is especially important in disease-prone areas, where timely immunisation is the first line of defence against common childhood infections [5].

The Expanded Programme on Immunisation (EPI), which was first implemented in 1978 and later expanded to include twelve antigens by 2013, has allowed Ghana to make notable progress in increasing its immunisation coverage [6]. To increase vaccine accessibility, the EPI provides both static and outreach immunisation services through a hierarchical structure that extends from the national level to local healthcare facilities [6]. To improve overall healthcare outcomes, immunisation programmes are combined with other child health interventions, such as growth monitoring and maternal education [7,8,9].

Despite these interventions, immunisation coverage in Ghana has experienced fluctuations over the years, influenced by socio-economic, geographic, and policy-related factors [7,8]. Historically, coverage improved significantly from 47% in 1988 to 79% in 2008 but later declined slightly to 77% in 2014 [7,8]. National trends also reveal regional variations; for instance, coverage in the Western Region dropped from 82% to 69% between 2008 and 2014 [3,4]. Regional disparities remain a key challenge, with urban–rural differences affecting vaccine uptake [3,4,8]. The Northern Region faces difficulties due to high mobility and sparse populations, while in the Upper East Region, more than three-quarters of children aged 12–23 months are fully vaccinated. In contrast, nearly 90% of children in the middle zone receive full immunisations [10].

Furthermore, several factors hamper immunisation coverage in Ghana. Poor knowledge about vaccines, limited access to immunising venues, financial constraints, long waiting times, and transport difficulties are significant barriers [10]. Additionally, socioeconomic disparities affect coverage, with children from poorer households and males being less likely to be fully immunised [11]. Access-related challenges are also notable in regions like Volta, where logistical constraints reduce vaccine uptake [12]. The Expanded Programme on Immunisation (EPI) plays a vital role in Ghana’s immunisation strategy but faces operational challenges that need to be addressed to improve efficiency [7,8,13]. Specific interventions, such as home visits and outreach services, have demonstrated potential in increasing vaccine coverage but are limited by supply side constraints [14].

Addressing regional disparities, socioeconomic inequalities, and logistical barriers is crucial to enhancing immunisation coverage in Ghana and ensuring that all children receive life-saving vaccinations, especially during the postnatal period [3,10,12]. The setting is a leading referral hospital, which provides critical postnatal immunisation services. The hospital’s Maternal and Child Health Clinic follows the EPI schedule when administering vaccines, ensuring that newborns receive BCG, oral polio (OPV), and other essential vaccines at birth and subsequent follow-up visits. These services are integrated with postnatal care (PNC) practices, such as growth monitoring, maternal counselling, and neonatal care, resulting in a comprehensive approach to early childhood health.

Postnatal immunisation services face several challenges, including a lack of awareness, misinformation, infrastructure constraints, socioeconomic barriers, and limited healthcare resources [15]. Misinformation about immunisation schedules can cause critical vaccinations to be delayed, exposing newborns to infection [15]. Vaccine stockouts can also be caused by infrastructure limitations in low-resource settings, as well as limited healthcare resources [6,16,17]. Socioeconomic barriers, such as financial constraints and cultural beliefs, also contribute to low vaccination rates [17]. Addressing these disparities necessitates targeted public health interventions. Some mothers exacerbate these limitations by believing vaccination begins 45 days after birth rather than immediately after delivery [15].

To give a thorough comprehension of the variables affecting postnatal immunisation practices in low-resource settings, we used the Socio-Ecological Model (SEM) as a theoretical framework [18,19]. It considers factors at the individual, interpersonal, organisational, community, and policy levels. Moreover, enhancing coverage necessitates a concerted effort at every socio-ecological level. Consequently, this prospective electronic survey study was conducted in the postnatal clinic of a tertiary referral national hospital to assess the postnatal immunisation practices. The findings could provide evidence-based interventions to improve immunisation coverage and recommend comprehensive approaches to dealing with contextual challenges.

## 2. Materials and Methods

### 2.1. Study Design

This study applied a quantitative, prospective cross-sectional design to evaluate postnatal vaccination practices among mothers who visited a tertiary hospital’s postnatal clinic [20]. The cross-sectional strategy was selected to collect real-time data on mothers’ adherence to vaccination regimens and associated behaviours during the immediate postpartum period, which is an important window for early childhood immunisation. This strategy allowed for the identification of trends and possible influencing factors across a defined timeframe, which was beneficial for understanding maternal and child health practices in an urban, resource-constrained healthcare context. The study followed the Strengthening the Reporting of Observational Studies in Epidemiology (STROBE) guidelines to guarantee scientific rigour, transparency, and reproducibility.

### 2.2. Study Setting and Population

The study was carried out in the postnatal clinic of an urban tertiary referral hospital. The clinic offers routine postnatal services, counselling, and medical care for mothers and infants after childbirth. The study included postnatal mothers aged 18 and up who attended the clinic for postnatal services, including immunisation and follow-up care, and who agreed to participate. We excluded mothers who were critically ill, unable to provide informed consent, or not the primary carer for the infant’s immunisation. Because the setting is a referral centre, these mothers came from a diverse range of socioeconomic and demographic backgrounds, making it an ideal place to describe various postnatal immunisation practices.

### 2.3. Sample and Sampling Techniques

We enrolled 184 postnatal mothers using convenience sampling, a non-probability sampling method that aims to capture a diverse group of mothers with different educational levels, employment statuses, and parity. To ensure relevant data, specific inclusion and exclusion criteria were established. Respondents were postnatal mothers who had given birth within the previous six weeks and were receiving a postnatal checkup at the clinic. They had to be literate in English or a widely spoken local language and capable of completing the questionnaire independently or with minimal assistance. Mothers with severe postpartum complications, as well as those who declined to participate or encountered language barriers, were excluded to ensure data accuracy and ethical consideration. The sample size for this survey was determined using power analysis, based on an expected medium effect size, a Type I error rate of 0.05, a desired statistical power of 0.80, and anticipated variability in the target population, ensuring sufficient sensitivity to detect statistically significant relationships between constructs [21].

### 2.4. Development of the Questionnaire

To ensure efficient data collection and accuracy, a structured electronic survey was designed and administered using Google Forms. This approach reduced the common errors associated with manual data entry while also improving respondents’ accessibility and ease of response. The questionnaire was carefully designed using constructs from the Socio-Ecological Model (SEM) and existing validated tools for assessing postnatal immunisation practices [19,22,23]. It was divided into three main sections in accordance with the study’s research objectives. The first section concentrated on demographic characteristics, gathering critical background data such as age, educational background, employment status, marital status, and parity. This data provides important context for understanding differences in postnatal immunisation practices across sociodemographic groups. The remaining sections assessed immunisation practices such as clinic attendance, schedule knowledge, and postpartum immunisation record-keeping. The questionnaire items were developed and organised using the Socio-Ecological Model (SEM), which captures influences at the individual, interpersonal, organisational, community, and policy levels to ensure a comprehensive assessment of factors influencing carers’ engagement with postnatal immunisation services [18,19].

The statements on the immunisation practices were evaluated on a five-point Likert scale (1 = Strongly Disagree, 2 = Disagree, 3 = Neutral, 4 = Agree, and 5 = Strongly Agree); disagreement was scored as 1 = strongly disagree and 2 = disagree, while agreement was scored as 4 = Agree to 5 = Strongly agree. Before collecting full-scale data, a pretest was conducted with a small sample of postnatal mothers (10% of the sample; 18) with similar characteristics different from the study main respondents to ensure that the questionnaire was clear, culturally relevant, and understandable to the respondents. The pre-test sample was excluded from the final analysis. This pre-test phase provided useful feedback on question wording, structure, and usability. Any ambiguities and difficulties in comprehension were addressed through revisions, increasing the reliability and validity of the final questionnaire used in the study.

### 2.5. Data Collection Procedures

Data was collected electronically using a Google Forms-based questionnaire, resulting in an efficient survey administration process. Respondents completed the survey on their own, using either the researchers’ electronic tablets or their personal mobile devices. To avoid response bias, researchers were available to explain survey items and clear up misunderstandings for mothers who needed assistance. To protect participants’ privacy and anonymity, all responses were de-identified, with no personally identifiable information recorded. The electronic format also improved data accuracy and security by automatically saving responses in a secure database. Participation in the study was entirely voluntary, and mothers were informed that they could opt out at any time.

### 2.6. Data Analysis

The collected data were analysed using IBM SPSS version 26 in a structured manner that combined descriptive and inferential statistical techniques. Initially, descriptive statistics were used to summarise participant characteristics, including means, standard deviations, frequencies, and percentages. This provided a comprehensive overview of demographic variables, allowing for a clearer understanding of the sample distribution. To describe the underlying structure of postnatal immunisation practices among postnatal mothers, factor analysis was performed using Principal Component Analysis. This required several key steps. First, the dataset’s factorability was assessed using the Kaiser-Meyer-Olkin (KMO) measure and Bartlett’s Test of Sphericity to ensure that the data were appropriate for factor analysis. The KMO test, ranging from 0 to 1, assesses sampling adequacy for factor analysis by examining small partial correlations between variables for compact patterns and determining underlying factors [24,25]. Communality is the percentage of a variable’s variance explained by extracted factors in factor analysis, which ranges from 0 to 1, indicating a good fit within the model [24,25].

The most significant factors influencing maternal postnatal immunisation practices were then identified through factor extraction using eigenvalues and variance percentages. Furthermore, communalities and scree plot analysis were used to assess the variance explained by extracted factors and visually determine the best number of factors to retain. Confirmatory Factor Analysis (CFA) is a statistical technique used to evaluate measurement models that represent hypotheses about relations between indicators and factors. We perform CFA to fit the model that emerged from this study [25]. We assessed Chi-square (χ^2^), Comparative Fit Index (CFI), Root Mean Square Error of Approximation (RMSEA), and Standardised Root Mean Square Residual (SRMR) as the model fit indices [25]. The results of this analysis were presented in the form of tables and graphs, which showed significant factor loadings, intercorrelations, and the distribution of respondents across the dataset.

### 2.7. Validity and Reliability Measures

Multiple validity and reliability measures were implemented to ensure the study’s findings were robust and accurate. Content validity was determined through expert review, in which maternal health specialists evaluated the questionnaire’s relevance in capturing postnatal immunisation practices, ensuring that the survey items were aligned with the study objectives and SEM, and comprehensively covered key postnatal immunisation activities. Furthermore, construct validity was assessed using factor analysis, which revealed distinct underlying dimensions associated with postnatal immunisation. To assess the reliability and validity of the scale measuring postnatal immunisation practices, internal consistency was evaluated using Cronbach’s alpha (α) and composite reliability (ω), while construct validity was examined through confirmatory factor analysis (CFA) with standardised loadings for each construct [26]. A reliability threshold of ≥0.7 was considered acceptable, indicating stable and consistent responses across different constructs [26]. These statistical approaches confirmed that the questionnaire accurately measured various aspects of postnatal immunisation, providing a solid foundation for interpreting the findings.

### 2.8. Ethical Considerations

Before data collection commenced, ethical approval was obtained from the Institutional Review Board (IRB) in the study setting. This approval ensured that the study adhered to international research ethics standards and prioritised the rights, dignity, and privacy of all respondents.

The goals, methods, possible risks, and advantages of the study were all explained in detail to the respondents. The online questionnaire’s initial pages included an information sheet and a form for informed consent. Before answering the primary survey questions, respondents had to voluntarily consent. To maintain confidentiality, all collected data was anonymised and securely stored, preventing unauthorised access or disclosure of respondent identities. Furthermore, voluntary participation was emphasised, with mothers informed of their right to withdraw from the study at any time without penalty. These safeguards ensured that participants felt comfortable taking the survey without being pressured or coerced, while adhering to ethical standards.

## 3. Results

### 3.1. Demographic Characteristics

Most respondents were between the ages of 27 and 30, with the youngest group at 18–22 (9.8%). They were married (47.3%), had at least two children (35.3%), had tertiary education (41.2%), and lived in urban cities (57.6%). Most infants were females (60.3%). The pregnancy rate was 63.1% planned, 31.5% unplanned, and 5.4% had unmet needs. Delivery took place primarily in health facilities (91.3%), with a high level of involvement in postnatal care. Vaccination data revealed that 40.8% of infants received their most recent vaccine at six weeks, with 41.8% receiving two doses. This emphasises the significance of vaccination programmes and regular postnatal care in improving maternal and child health. Refer to Table 1 for details.

### 3.2. Practices of Postnatal Immunisation Services

We observed that mothers generally followed recommended vaccine schedules, with a mean score of 3.86. However, there was a variation in parental compliance. Parents felt they were given comprehensive resources on postnatal immunisation education and vaccine-preventable diseases, with a mean score of 3.79. They also felt that the services were adequate in meeting cultural and linguistic needs. However, satisfaction with comfort and privacy, as well as healthcare providers’ professionalism and expertise during immunisation sessions, was rather moderate. Parents did not keep good records of their infants’ immunisations. Parents actively sought information and support from healthcare providers, but some declined to recommend the services to others. See Table 2.

### 3.3. Factor Analysis on Practices of Postnatal Immunisation Services

We determined the factorability of the data by performing Kaiser Mayer-Olkin (KMO), and Bartlett’s Test of Sphericity and Variance. The KMO value of 0.865 and Bartlett’s Test of Sphericity of 1956.952 indicate that the data is appropriate for factor analysis. Thus, the data was appropriate for identifying underlying factors influencing postnatal immunisation practices.

#### 3.3.1. Communalities

Communalities in Table 3 demonstrates the results of a Principal Component Analysis (PCA), indicating how well each statement correlates with the extracted components related to postnatal immunisation practices. Higher extraction values indicate more robust associations with the underlying factors. We discovered that parental satisfaction with postnatal immunisation services is significantly linked to positive experiences. High communalities include comfort and privacy during immunisation sessions, understanding the importance of immunisation, appointment reminder communication methods, and healthcare provider professionalism. Inadequate adherence to vaccine schedules, access to immunisation education, seeking information from healthcare providers, and guidance on managing vaccine side effects are examples of suboptimal postnatal immunisation practices. The findings indicate that improvements in education, culturally sensitive services, and accessibility could increase immunisation rates and parental confidence.

#### 3.3.2. Principal Component Analysis: Total Variance Explained

We then performed Principal Component Analysis (PCA), the results of which are shown in Table 4. Table 4 shows the distribution of variance explained by each extracted component in postnatal immunisation practices. The findings shed light on the most important factors influencing postnatal immunisation schedule compliance. Our analysis shows that adhering to the recommended vaccination schedule is the most important factor influencing postnatal immunisation practices and outcomes, accounting for 56.41% of the total variance. This ensures infants receive vaccinations on time, lowering the risk of vaccine-preventable diseases. Satisfaction with communication is the second-most influential factor, accounting for 8.34% of the variance. Effective communication strategies, parental engagement, and follow-up reminders are critical to vaccination uptake. These first two components (adherence to schedules, and effective communication during session) accounted for a cumulative variance of 64.75%, indicating the significant factors influencing immunisation practices.

The remaining variables explained only a small portion of the total variance in postnatal immunisation practices in the setting. These suboptimal factors include access to comprehensive resources, understanding the importance of immunisation, cultural and linguistic considerations, comfort and privacy, healthcare provider professionalism, availability of adequate vaccine information, convenient service location and hours, immunisation record maintenance, support for managing side effects, seeking information and support from healthcare providers, and recommending immunisation services. This demonstrates a concerning trend of ineffective immunisation practices in these areas.

#### 3.3.3. Scree Plot on Practices of Postnatal Immunisation

**Figure 1:**
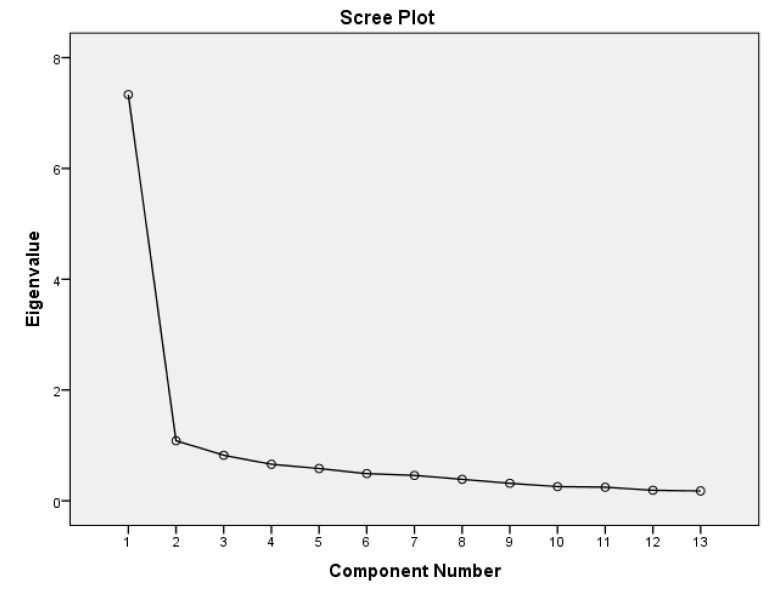
Scree plot on practices of postnatal immunisation services.

In Figure 1, the scree plot evaluates the variance explained by each principal component in a dataset by graphically representing its eigenvalues. The change from significant to minimal components that explain variance is indicated by the “elbow” point at the second component. The first two components are the dominant and most significant variance, as indicated by the plot’s sharp decline in eigenvalues from the first two to the remaining components. This supports the Principal Component Analysis (PCA) findings reported in Table 4.

### 3.4. Confirmatory Factor Analysis on Postnatal Immunisation Practices

A confirmatory factor analysis (CFA) was conducted to examine the hypothesised two-factor model of practices related to postnatal immunisation. The model was estimated using the Maximum Likelihood (ML) estimation method. The chi-square test demonstrated a non-statistically significant result, χ^2^(34) = 91.333, *p* < 0.001.

#### 3.4.1. Model Fits Indices

Goodness-of-fit indices indicated an acceptable model fit. Specifically, the Comparative Fit Index (CFI = 0.945), Tucker–Lewis Index (TLI = 0.927), and Incremental Fit Index (IFI = 0.946) were all close to or above the recommended threshold of 0.95. The Root Mean Square Error of Approximation (RMSEA) was 0.071; <0.08, suggesting an acceptable fit. The Standardised Root Mean Square Residual (SRMR) value was 0.042, falling within acceptable limits (<0.08).

#### 3.4.2. Factor Loadings and Significance on the Practices of Postnatal Immunisation

The confirmatory factor analysis revealed two latent constructs (Table 5): immunisation adherence and effective communication. Immunisation adherence made a considerable contribution to the model, with standardised estimates ranging from 0.683 to 1.054, while effective communication standardised loadings ranged from 0.859 to 1.000.

#### 3.4.3. Reliability and Validity on the Practices of Postnatal Immunisation

**Figure 2:**
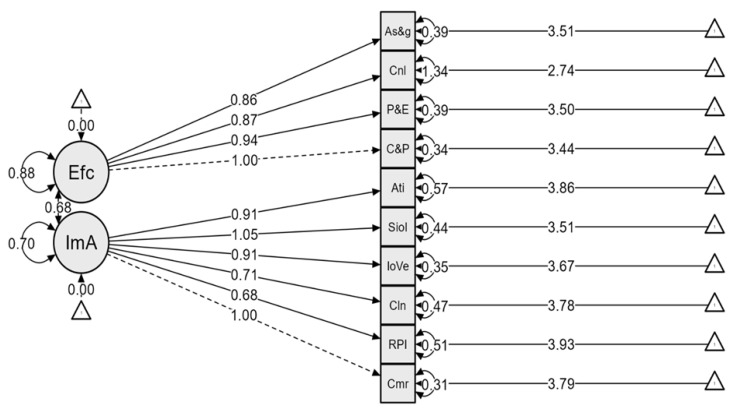
Confirmatory factor model plot on the two-factor model for postnatal immunisation practices.

The scale demonstrated strong internal consistency. Cronbach’s alpha coefficients were 0.823 for effective communication and 0.877 for adherence to immunisation schedules. Composite reliability (*ω*) for effective communication was good: 0.832, and that of adherence to immunisation schedule was 0.877. The overall reliability was excellent (*α* = 0.909, *ω* = 0.918). Figure 2 presents the standardised CFA model showing clear and appropriate loadings of observed variables onto their respective latent constructs.

## 4. Discussion

We investigated the practices of postnatal immunisation services in a tertiary facility in a low-resource setting. The findings show that postnatal mothers’ good immunisation practices were influenced by their adherence to immunisation schedules, significant satisfaction with communication, the level of comfort and privacy provided, and professionalism demonstrated by healthcare providers. However, there were some concerning trends in mothers’ suboptimal practices regarding postnatal immunisation. These suboptimal practices include limited access to comprehensive resources, poor immunisation record maintenance, insufficient support for managing vaccine side effects, a low desire to seek information and support from healthcare providers, and mothers’ unwillingness to recommend immunisation services to their peers. Most of these findings are consistent with existing studies, while some are context specific. The final two-factor model had consistent findings with the SEM that was deployed as the theoretical framework [18,19].

The study identified a high adherence rate to postnatal immunisation schedules among mothers, occurring within a context where structured immunisation programmes are designed to ensure timely vaccine administration [2,27]. Anecdotally, some interrelated factors likely contribute to this high adherence, including robust public health policies, effective communication strategies, and increased parental awareness of the importance of immunisation. These findings align with studies conducted in Northwest Ethiopia, which emphasise that adherence rates tend to be higher in regions with well-established healthcare systems and structured immunisation programmes [28,29]. This further highlight that community-driven awareness campaigns and routine follow-up mechanisms play a critical role in ensuring high immunisation adherence.

Conversely, a systematic review reported socioeconomic and gender variables as the barriers and facilitators to immunisation in Sub-Saharan Africa [30]. Perhaps the low adherence rates are predominant in settings with weaker healthcare infrastructures, illustrating how disparities in system organisation impact vaccine uptake. Similarly, a study from Nigeria identified logistical challenges, including a lack of accessibility to healthcare centres and poor record-keeping, as barriers to immunisation adherence 22. These contrasts highlight the importance of strong health system governance, efficient service delivery, and parental engagement in maintaining high immunisation rates. Reports indicate that timely short message service (SMS) reminders to mothers enhance immunisation rates in Nigeria [31].

One of the key factors contributing to the observed high adherence in the current study is the effective implementation of communication and reminder systems by healthcare providers [31]. These systems, which include automated reminders, outreach by community health workers, and structured follow-up visits, have been shown to improve parental compliance with vaccination schedules [28,30,31]. Additionally, public health education campaigns emphasising the risks of vaccine-preventable diseases further reinforce the need for timely immunisation. To maintain immunisation adherence, healthcare systems should improve reminder systems, increase accessibility, provide privacy and comfort during immunisation sessions, show professionalism from the healthcare providers, and expand health education programmes [28,32]. Future research should explore the specific barriers and facilitators of adherence in regions with lower vaccination rates and identify context-specific interventions to improve postnatal immunisation practices.

Despite the above satisfactory immunisation practices, we identified several factors associated with suboptimal postnatal mothers’ immunisation practices, including poor immunisation record-keeping, limited knowledge of vaccine side effects, inadequate provider support in managing side effects, and reluctance to recommend immunisation to other mothers. These findings highlight critical gaps in postnatal immunisation adherence, which may undermine the effectiveness of routine immunisation programmes. Poor immunisation recordkeeping among mothers suggests potential barriers to tracking vaccination status, which can lead to missed or delayed vaccinations. This finding aligns with earlier research that emphasised the importance of adhering to standard immunisation schedules, which should begin during the postnatal period [5,17]. Inadequate record keeping can contribute to incomplete immunisation, increasing the risk of vaccine-preventable diseases. Strengthening record-keeping practices through digital tracking systems and maternal education on documentation may help mitigate this issue [30,31].

Limited awareness of vaccine side effects among postnatal mothers further complicates these suboptimal immunisation practices. Mothers who lack knowledge about expected post-vaccination reactions may misinterpret mild side effects as severe, leading to unnecessary concerns and vaccine hesitancy [33,34]. Possibly, the fact that most postnatal immunisation vaccines, including BCG, polio, and DPT, cause some mild reactions could explain this finding. However, if mothers are not well informed about these expected side effects, they may be less likely to return for subsequent doses or recommend immunisations to others [34]. As a result, providing clear, culturally sensitive education on vaccine side effects could enhance maternal confidence and immunisation adherence. Additionally, the lack of sufficient provider support in managing vaccine side effects may contribute to dissatisfaction and hesitancy among mothers. Research indicates that suboptimal immunisation practices, including inadequate provider engagement, can lead to weak immune responses and lower vaccine acceptance [35]. Thus, ensuring that healthcare providers offer comprehensive guidance on side effect management, along with follow-up support, is essential to improving maternal trust in immunisation services.

The reluctance of some mothers to recommend immunisations to their peers underscores the broader impact of vaccine hesitancy within communities. This finding is consistent with earlier research in Nigeria, which reported decreased utilisation of immunisation due to poor maternal knowledge [36]. As highlighted by Mihret [27], in an Ethiopian study, the low knowledge threshold of mothers thwarted efforts towards improved postnatal immunisation. This is a worrying situation, as mothers who feel unsupported or lack sufficient information may discourage others from participating in immunisation programmes, reducing overall coverage. As such, strategies, including peer education programmes and community-driven advocacy, could help reinforce positive attitudes towards vaccination. To address these challenges, targeted interventions should focus on enhancing maternal education, strengthening healthcare provider communication, and improving immunisation tracking mechanisms. Future research should explore the long-term impact of these interventions on immunisation uptake and assess the effectiveness of different communication strategies for addressing vaccine hesitancy among postnatal mothers.

### 4.1. Strengths and Limitations

This study provides a comprehensive analysis of postnatal immunisation practices in low-resource settings, highlighting both excellent and suboptimal practices. It provides context-specific insights into healthcare planning and policy implementation, highlighting unique challenges faced by mothers and healthcare providers in resource-limited settings. The study aligns with global evidence on immunisation adherence, emphasising the importance of strong healthcare governance, effective communication strategies, and parental education. It offers practical recommendations for improving maternal engagement in immunisation programmes and holds significant potential for policy impact. However, the study’s focus on a single tertiary healthcare facility limits its generalisability to other regions with different healthcare infrastructures and socio-economic conditions. Additionally, the cross-sectional design captures immunisation practices at a single point in time, limiting our understanding of broader systemic barriers. Furthermore, the utilisation of convenience sampling may limit the generalisability of results, as participants who attended the clinic during the data collection period may not fully represent all postnatal mothers.

### 4.2. Future Directions

This study emphasises the need for additional research to identify context-specific interventions that improve postnatal immunisation adherence. It advocates a better understanding of socio-cultural and systemic factors that contribute to suboptimal practices in low-resource settings, as well as the development of innovative technological solutions such as mobile health applications and electronic immunisation registries. It also emphasises the importance of conducting targeted research on vaccine hesitancy among postnatal mothers, specifically looking at how peer education programmes, culturally sensitive campaigns, and community engagement affect maternal confidence. Finally, it suggests evaluating the effectiveness of healthcare provider training for improving postnatal immunisation practices. Furthermore, future research should consider a multi-site approach, longitudinal data collection, and exploring systemic barriers and facilitators in greater depth.

### 4.3. Implications for Practice

The study proposes several strategies for improving postnatal immunisation services in tertiary healthcare facilities, particularly in low-resource settings. These include strengthening record-keeping systems, improving communication and support, increasing community engagement through peer education programmes, optimising immunisation outcomes, and incorporating SMS-based reminders in postnatal care services. These measures are intended to reduce missed or delayed vaccinations, address maternal concerns about vaccine side effects, and promote trust in immunisation services. Furthermore, ensuring privacy, comfort, and professionalism during immunisation sessions can boost satisfaction and compliance. To reduce vaccine-preventable diseases and improve maternal and child health outcomes, the healthcare systems could consider implementing these strategies.

Our findings might help us develop evidence-based policies and guidelines for postnatal immunisation practices. The policy should point out the importance of good communication, culturally sensitive care, and service accessibility. As a result, health policymakers might use this research to improve immunisation education, integrate reminder systems, and train workers on respectful, maternity-centred care. Furthermore, the study’s emphasis on caregiver experience could contribute to the development of contextually relevant policies that address structural barriers in similar urban and resource-constrained contexts in low- and middle-income countries.

## 5. Conclusions

We observed that comfort, privacy, structured programmes, community awareness initiatives, and effective communication all have an impact on postnatal immunisation practices. High satisfaction with experience promotes positive maternal engagement and encourages continuous participation. However, issues such as inadequate support and record-keeping gaps must be addressed. Improving provider communication, implementing digital tracking systems, and enhancing maternal education could all help increase immunisation coverage and reduce vaccine-preventable diseases. Future research could examine the barriers and facilitators of developing context-specific interventions for populations with low adherence rates.

## Figures and Tables

**Table 1:** Demographic characteristics of the respondents.

Variable	Category	Freq.	%
Age (years)	18–22	18	9.8
	23–26	41	22.3
	27–30	56	30.4
	31–34	29	15.8
	35–38	25	13.6
	39–42	12	6.5
	Above 42	3	1.6
Marital Status	Single	54	29.3
	Married	87	47.3
	Consensual Union	7	3.8
	Never married	3	1.7
	Divorced	33	17.9
Religion	Christian	142	77.2
	Islam	42	22.8
Number of Children	1	60	32.6
	2	65	35.3
	3	38	20.7
	4	18	9.8
	5	3	1.6
Occupational Status	Employed (full-time)	60	32.6
	Employed (part-time)	29	15.7
	Self-employed	68	37.0
	Unemployed	27	14.7
Education attainment	No education	14	7.6
	Basic education	15	8.2
	Secondary education	45	24.5
	Tertiary education	76	41.2
	Postgraduate	11	6.0
	Vocational	23	12.5
Partner’s level of education	No education	7	3.8
	Basic education	7	3.8
	Secondary education	29	15.8
	Tertiary education	102	55.4
	Postgraduate	25	13.6
	Vocational	14	7.6
Residence	Urban	106	57.6
	Peri-Urban	35	19.0
	Rural	43	23.4
Distance from your home to the postnatal clinic	Below 30 min	33	17.9
	About 30 min to one hour	70	38.1
	Above one hour	81	44.0
Mode of transportation	On foot	27	14.7
	By car	157	85.3
Gender of the infant	Male	73	39.7
	Female	111	60.3
Pregnancy status	Planned	116	63.1
	Unplanned	58	31.5
	Unmet needs	10	5.4
Place of delivery	Home	8	4.3
	Health facilities	168	91.3
	TBA	8	4.4
Number of PNC visits	One time	27	14.7
	Two time	84	45.7
	More than two times	73	39.6
The last vaccine the infant received	At birth	18	9.8
	Six weeks	75	40.8
	Ten weeks	27	14.7
	Fourteen weeks	53	28.7
	Not sure	11	6.0
Mother’s tetanus-diphtheria	No dose received	7	3.8
	1 dose received	42	22.8
	2 doses received	77	41.8
	3 doses received	58	31.6

**Table 2:** Descriptive statistics on the practices of postnatal immunisation services.

Statements	Mean	Mode	Std. Deviation	Percentiles			IQR
				25	50	75	
I adhere to the recommended vaccine schedule provided by the healthcare professional in ensuring that my infant receive vaccination at the appropriate times.	3.857	4	1.075	4	4	5	1
I am satisfied with the communication methods used to remind me on fellow ups for postnatal immunisation appointments	3.698	4	1.139	3	4	4	1
I am offered comprehensive resource on postnatal immunisation education and vaccine preventable diseases	3.788	4	1.009	4	4	4	0
I understand the importance of postnatal immunisation for the health and well-being of my infant.	3.757	4	1.098	4	4	4	0
Postnatal immunisation services adequately address my cultural and linguistic needs.	3.778	4	0.907	3	4	4	1
I feel satisfied with the level of comfort and privacy provided during postnatal immunisation sessions for me and my infant.	3.439	4	1.107	2	4	4	2
I am satisfied with the professionalism and expertise demonstrated by healthcare providers delivering postnatal immunisation services.	3.497	4	1.079	2	4	4	2
Postnatal immunisation services offer me adequate information on potential vaccine side effects and how to manage them	3.672	4	0.967	3	4	4	1
The location and hours of operation is convenient for my infant’s postnatal immunisation services.	2.735	2	1.417	2	2	4	2
I maintain records of my infant’s postnatal immunisations including the types of vaccines received and the date administered in the vaccination booklets provided by the hospital.	3.825	4	0.835	4	4	4	0
I receive adequate support and guidance for managing any adverse reactions or side effects.	3.513	4	1.019	2.5	4	4	1.5
I seek information and support from healthcare providers at the hospital regarding postnatal immunisations including questions or concerns about the immunisation process or vaccine safety.	3.508	4	1.109	2	4	4	2
I recommend postnatal immunisation services to other parents/caregivers based on my experience.	3.926	4	0.919	4	4	5	1

Statements evaluated on a five-point Likert scale (1 = Strongly Disagree, 2 = Disagree, 3 = Neutral, 4 = Agree, and 5 = Strongly Agree).

**Table 3:** Communalities on the practices of postnatal immunisation services.

Statement: Good Postnatal Immunisation Practices	Initial	Extraction
I am satisfied with the communication methods used to remind me on fellow ups for postnatal immunisation appointments	1.000	0.718
I understand the importance of postnatal immunisation for the health and well-being of my infant.	1.000	0.746
I feel satisfied with the level of comfort and privacy provided during postnatal immunisation sessions for me and my infant.	1.000	0.756
I am satisfied with the professionalism and expertise demonstrated by healthcare providers delivering postnatal immunisation services.	1.000	0.706
Statement: Inadequate Postnatal Immunisation Practices	Initial	Extraction
I adhere to the recommended vaccine schedule provided by the healthcare professional in ensuring that my infant receive vaccination at the appropriate times.	1.000	0.668
I am offered comprehensive resource on postnatal immunisation education and vaccine preventable diseases	1.000	0.682
Postnatal immunisation services adequately address my cultural and linguistic needs.	1.000	0.529
Postnatal immunisation services offer me adequate information on potential vaccine side effects and how to manage them.	1.000	0.647
The location and hours of operation is convenient for my infant’s postnatal immunisation services.	1.000	0.625
I maintain records of my infant’s postnatal immunisations including the types of vaccines received and the date administered in the vaccination booklets provided by the hospital.	1.000	0.536
I receive adequate support and guidance for managing any adverse reactions or side effects.	1.000	0.647
I seek information and support from healthcare providers at the hospital regarding postnatal immunisations including questions or concerns about the immunisation process or vaccine safety.	1.000	0.655
I recommend postnatal immunisation services to other parents/caregivers based on my experience.	1.000	0.503

Extraction method: Principal Component Analysis.

**Table 4:** Total variance explained: practices of postnatal immunisation services.

Component	Initial Eigenvalues			Extraction Sums of Squared Loadings		
	Total	% of Variance	Cumulative%	Total	% of Variance	Cumulative%
Adherence to schedule	7.333	56.407	56.407	7.333	56.407	56.407
Satisfaction with postnatal immunisation communication	1.084	8.338	64.745	1.084	8.338	64.745
Offered comprehensive resources.	0.822	6.324	71.069			
Understand the importance of immunisation	0.659	5.068	76.137			
Immunisation adequately addresses my culture and linguistic needs	0.582	4.480	80.617			
Satisfaction with level of comfort and privacy	0.490	3.770	84.387			
Satisfaction with professionalism	0.459	3.530	87.916			
Immunisation services offer me adequate information	0.387	2.980	90.897			
Convenient location and hours of operation	0.315	2.423	93.320			
Maintain immunisation records of my infant	0.256	1.973	95.293			
Receive adequate support and guidance	0.244	1.878	97.171			
Seek information and support from healthcare providers	0.190	1.461	98.632			
Recommendation of postnatal immunisation services	0.178	1.368	100.000			

Extraction Method: Principal Component Analysis.

**Table 5:** Factor loadings and significance.

						95% Confidence Interval	
Factor	Indicator	Estimate	Std. Error	z-Value	*p*	Lower	Upper
Immunisation Adherence	Comprehensive resources	1.000	0.000			1.000	1.000
	Recommend postnatal immunisation	0.683	0.075	9.114	<0.001	0.536	0.830
	Cultural needs	0.706	0.073	9.652	<0.001	0.562	0.849
	Information on Vaccine effects	0.913	0.073	12.509	<0.001	0.770	1.056
	Seeking information on immunisation	1.054	0.083	12.622	<0.001	0.890	1.217
	Adherence to immunisation	0.912	0.084	10.808	<0.001	0.747	1.078
Effective communication	Comfort and privacy	1.000	0.000			1.000	1.000
	Professionalism and expertise	0.938	0.072	13.012	<0.001	0.796	1.079
	Convenient location	0.867	0.105	8.231	<0.001	0.660	1.073
	Adequate support and guidance	0.859	0.069	12.488	<0.001	0.724	0.994

## Data Availability

The data presented in this study are available upon request from the corresponding author due to ethical reasons. Access to the data may be considered on a case-by-case basis and can be obtained by contacting the corresponding author at emmanuel.korsah1@wits.ac.za.

## Abbreviations

The following abbreviations are used in this manuscript:

EPI	Expanded Programme on Immunisation
BCG	Bacillus Calmette-Guérin
OPV	Oral polio vaccine
DPT	Diphtheria, pertussis, tetanus
PNC	Postnatal care
SEM	Socio-Ecological Model
STROBE	Strengthening the Reporting of Observational Studies in Epidemiology
KMO	Kaiser-Meyer-Olkin
CFA	Confirmatory Factor Analysis
CFI	Comparative Fit Index
RMSEA	Root Mean Square Error of Approximation
SRMR	Standardised Root Mean Square Residual
PCA	Principal Component Analysis
TLI	Tucker–Lewis Index
IFI	Incremental Fit Index
